# Medical Items

**Published:** 1908-02

**Authors:** 


### MEDICAL ITEMS

---

ELIMINATIVE TREATMENT. It cannot be denied that falty elemination of the products of metabolism, is a common predisposing cause of disease. Such cases, regardless of name, are promptly relieved by stimulating elimination through the kidneys. There is no better renal eliminant than Alkalithia.

---

### THE COUGHS FOLLOWING GRIP.

---

Dr. John McCarty (Louisville Medical College), in giving his personal experience with this condition, writes as follows: "Ten years ago, I had the grip severely, and every winter until 1902, my cough was almost intolerable. During January 1902, I procured a supply of Antikamnia and Codeine Tablets and began taking them for my cough, which had distressed me all winter, and as they gave me prompt relief, I continued taking them with good results. Last fall I again ordered a supply of Antikamnia and Codeine Tablets and I have taken them regularly all winter and have coughed but very little. I take one tablet every three or four hours and one on retiring. They not only stop the cough, but make expectoration easy and satisfactory. The best results are obtained by allowing the tablet to dissolve slowly in the mouth before swallowing."

---

President Manuel Amador, of Panama, tells this little tale of a certain Cuban millionaire:

"An unfortunate man once obtained access to this millionaire and started to lay before him his woes. He depicted his wretched poverty in most vivid colors. Indeed, so graphic was the man's sad story that the millionaire felt himself affected as he had never been before. With tears in his eyes he summoned his servant and in a quavering voice, said:

"John, put this poor fellow out. He is breaking my heart."

*January Everybody's*

### RESPIRATORY TRACT:

### AFFECTIONS, SYMPTOMS AND TREATMENT.

BY DR. ARTHUR B. SMITH, SPRINGFIELD, O.

---

The average physician is frequently vexed in finding a condition which resists his best efforts to bring about a cure. This holds good in almost every disease at some time or other, but particularly in affections of the respiratory tract, where there may be a great variety of symptoms in several cases of the same disease.

Almost every physician has had some favorite prescription for coughs, bronchitis, laryngitis, etc., which he uses until suddenly it seems to lose its efficacy—why, no one knows. Then another remedy takes its place until it, too, fails to give the desired result. It is rarely that one finds a cough remedy which will be consistently good in the majority of cases. Theoretically there appears to be a well-founded objection to the use of cough syrups in general, but nevertheless, there are times when nothing else gives satisfaction; therefore, the physician pins his faith to that remedy from which he and his patients derive the most good. It is not always easy to find such a remedy, but when it is once found, it is equally difficult to dispense with, and often the physician is almost compelled to resort to a routine treatment. In such cases, of course, he wants the best.

There are constantly being placed on the market new formulas for affections of the air passages. Some of these formulas are of undoubted benefit in some cases, but usually it will be found that the results are far from satisfactory. Many of them cannot be taken when there is any gastric complication as is sometimes the case, because of consequent nausea and vomiting. Others seem almost invariably to act as cardiac depressants and are highly objectionable for that reason.

In phthisical patients the well known lack of appetite and intolerance of various foods render it imperative to give remedies which will not in any way interfere with the digestive functions, while at the same time controlling or alleviating the cough and other distressing conditions.

Some time ago my attention was called to a preparation composed of a solution of heroin in glycerine, combined with expectorants, called Glyco-Heroin (Smith.) Each teaspoonful of this preparation contains one-sixteenth grain of heroin by accurate dosage. It is of agreeable flavor, therefore easy to administer to children, for whom the dose can be easily reduced with any liquid, or by actual measurement. It possesses many advantages not shown by any other preparation I have used, and has none of their disagreeable features.

In citing some of the cases treated with this remedy, I shall not go into a minute description of any case, but briefly state the conditions which existed and the results obtained, which were uniformly good.

Case 1. S. B., aged 16. Caught a severe cold while traveling. This developed into an unusually severe attack of bronchitis with mucous rales, pain cough and some slight fever. Prescribed Glyco-Heroin (Smith) one teaspoonful every two hours, decreased to every three hours. After a few doses were taken there was a decided improvement, the respirations were slower and deeper, the expectoration accomplished with more ease and the temperature normal. In a few days the patient was practically well and able to return to school. No medicine except Glyco-Heroin (Smith) was given and the results from its use were excellent.

Case 2. W. L., aged 31. Acute bronchitis. Painful cough, with difficult expectoration, particularly when in reclining posture. Glyco-Heroin (Smith) in teaspoonful doses every three hours gave speedy relief and a cure was effected in a few days.

Case 3. B. E., aged 26. Severe bronchitis accompanying an attack of influenza. Various remedies were tried in this case, with negative results, until Glyco-Heroin (Smith) was given in teaspoonful doses every three hours. In a short time decided relief was obtained and the cough stopped permanently.

Case 4. R. L., aged 6. Capillary bronchitis with pains over chest, cough and difficult expectoration. Glyco-Heroin (Smith) administered 15 drops every three hours. After taking a few doses, the condition was much improved, and a speedy return to perfect health followed.

Case 5. W. H., aged 5. Whooping cough. Spasmodic paroxysms of coughing, sometimes being so severe as to cause vomiting. Tenacious mucous was present, requiring great expulsive effort to loosen it. There was little fever, but the patient was much prostrated and weakened by the cough. Glyco-Heroin (Smith) was given in 10 drop doses every two hours with good results. This was combined with hygienic treatment, the patient being given as much fresh air as possible. In a few days, the condition was ameliorated, the cough under fair control, expectoration more free and easier to raise, and convalescence uneventful. The case was discharged cured and there were no unpleasant sequelae, the patient at present being in prefect health.

---

### THE MILKY WAY.

To boil or not to boil, that is the question:
Whether 'tis nobler, recklessly to swallow
The germs and toxins of raw lacteal blend,
Or to take arms against this sea of microbes,
And, by parboiling, end them? To pasteurize—
No more, the doctors cry, nor sterilize,
For thus we slay the thousand healthful germs,
That milk is heir to; 'tis consummation
Devoutly to avoid. Thus would they lull
Our fears to sleep. We dream; aye, there's the rub,
For in that sleep of dreams, what rude alarms
Awaken us! The milkman's morning call
Disturbs our rest. There stands the milk,
A bottled menace unto human life.
Yet must we pay for quarts of bev'rage which,
With germs benign, is mixed the contumely
Of rural scorn for hygienic laws,
And city dealers' dash of formalin,
Put in to lower the count bacterial.
Inspection does make cowards of us all;
And thus the native hue of bovine milk
Is sicklied o'er with the pale cast of doubt.
From Cow to Milch Goat let us turn our thoughts,
To Nanny, late despised, but now the queen
Of rediscovered country, that fair realm
Of capriculture, whence her lacteal yield
Gives health and strength from infancy to age.

—A Caprine Soliloquy by Julia Harries Bull in *Good Housekeeping.*

